# Late initiation of renal replacement therapy is associated with worse outcomes in acute kidney injury after major abdominal surgery

**DOI:** 10.1186/cc8147

**Published:** 2009-10-30

**Authors:** Chih-Chung Shiao, Vin-Cent Wu, Wen-Yi Li, Yu-Feng Lin, Fu-Chang Hu, Guang-Huar Young, Chin-Chi Kuo, Tze-Wah Kao, Down-Ming Huang, Yung-Ming Chen, Pi-Ru Tsai, Shuei-Liong Lin, Nai-Kuan Chou, Tzu-Hsin Lin, Yu-Chang Yeh, Chih-Hsien Wang, Anne Chou, Wen-Je Ko, Kwan-Dun Wu

**Affiliations:** 1 Division of Nephrology, Department of Internal Medicine, Saint Mary's Hospital, 160 Chong-Cheng South Road, Lotung 265, I-Lan, Taiwan; 2 Division of Nephrology, Department of Internal Medicine, National Taiwan University Hospital, 7 Chung-Shan South Road, Taipei 100, Taiwan; 3 Division of Nephrology, Department of Internal Medicine, National Taiwan University Hospital Yun-Lin Branch, No.579, Sec. 2, Yunlin Rd., Douliu City, Yunlin County 640, Taiwan; 4 National Center of Excellence for General Clinical Trial and Research, National Taiwan University Hospital, 7 Chung-Shan South Road, Taipei 100, Taiwan; 5 Department of Surgery, National Taiwan University Hospital, 7 Chung-Shan South Road, Taipei 100, Taiwan; 6 Department of Anesthesiology, National Taiwan University Hospital, 7 Chung-Shan South Road, Taipei 100, Taiwan

## Abstract

**Introduction:**

Abdominal surgery is probably associated with more likelihood to cause acute kidney injury (AKI). The aim of this study was to evaluate whether early or late start of renal replacement therapy (RRT) defined by simplified RIFLE (sRIFLE) classification in AKI patients after major abdominal surgery will affect outcome.

**Methods:**

A multicenter prospective observational study based on the NSARF (National Taiwan University Surgical ICU Associated Renal Failure) Study Group database. 98 patients (41 female, mean age 66.4 ± 13.9 years) who underwent acute RRT according to local indications for post-major abdominal surgery AKI between 1 January, 2002 and 31 December, 2005 were enrolled The demographic data, comorbid diseases, types of surgery and RRT, as well as the indications for RRT were documented. The patients were divided into early dialysis (sRIFLE-0 or Risk) and late dialysis (LD, sRIFLE -Injury or Failure) groups. Then we measured and recorded patients' outcome including in-hospital mortality and RRT wean-off until 30 June, 2006.

**Results:**

The in-hospital mortality was compared as endpoint. Fifty-seven patients (58.2%) died during hospitalization. LD (hazard ratio (HR) 1.846; *P *= 0.027), old age (HR 2.090; *P *= 0.010), cardiac failure (HR 4.620; *P *< 0.001), pre-RRT SOFA score (HR 1.152; *P *< 0.001) were independent indicators for in-hospital mortality.

**Conclusions:**

The findings of this study support earlier initiation of acute RRT, and also underscore the importance of predicting prognoses of major abdominal surgical patients with AKI by using RIFLE classification.

## Introduction

Acute kidney injury (AKI) is a common problem in critically ill patients, with a reported incidence of 1 to 25% and a poor prognosis [1,2]. Postoperative AKI is one of the most serious complications in surgical patients [3]. The risk factors of postoperative AKI include emergent surgery [4], exposure to nephrotoxic drugs, hypotension, hypovolemia, hypothermia, inflammatory response to surgery [5,6], and cardiac dysfunction [3]. On the other hand, hospital-acquired infection also contributes to the development of AKI in patients who receive emergent abdominal surgery. The abdominal compartment syndrome, which develops after sustained and uncontrolled intra-abdominal hypertension and may result in AKI or mortality, is being increasingly observed in the general surgical population [7]. Thus it was assumed that abdominal surgery is probably associated with an increased likelihood of developing AKI.

The appropriate timing of renal replacement therapy (RRT) initiation in patients with AKI has been under debate for a long time. From the view point of an early renal support strategy, the goal of early RRT is to maintain solute clearance and fluid balance to prevent subsequent multi-organ damage, while waiting for the recovery of renal function [8]. Although a meta-analysis by Seabra and colleagues [9] revealed a beneficial effect of early initiation of RRT, the benefits of early acute dialysis remain controversial [10-12]. The aim of the present study was to evaluate whether the timing of RRT affected the in-hospital mortality rate in patients with AKI after major abdominal surgery.

## Materials and methods

### Study populations

This study was based on the National Taiwan University Surgical ICU Associated Renal Failure (NSARF) Study Group database. The database was constructed for quality and outcome assurance in one medical center (National Taiwan University Hospital, Taipei, Northern Taiwan) and its three branch hospitals in different cities. Since 2002, the database recruited all patients requiring RRT during their intensive care unit (ICU) stay, and prospectively collected data in these four hospitals [13-15]. From January 2002 to December 2005, adult patients who underwent major abdominal surgery with postoperative AKI requiring RRT in ICU were enrolled into this multicenter prospective observational study. Exclusion criteria included patients aged less than 18 years, patients with an ICU stay of less than two days, patients who started dialysis before surgery, patients who didn't undergo abdominal surgery, or patients who underwent renal transplantation. Those enrolled were treated by the same team of physicians and nurses, and followed until 30 June, 2006. Surgical procedures were considered major if the length of hospital stay for patients in a given diagnosis-related group exceeded two days [15-18]. Informed consent was waived because there was no breach of privacy and it did not interfere with clinical decisions related to patient care. Approval for this study was obtained from the Institutional Review Board of National Taiwan University Hospital (No. 31MD03).

### Patient information and data collection

The demographic data, comorbid diseases, types of surgery and RRT, as well as the indications for RRT were documented. The biochemistry data such as complete blood cell count, blood urea nitrogen (BUN), serum creatinine (sCr), glomerular filtration rate (GFR), serum albumin, and serum potassium (sK^+^) were recorded upon ICU admission and RRT initiation. Severity scores including Glascow Coma Scale (GCS) score, Acute Physiology and Chronic Health Evaluation II (APACHE II) [19] score, and Sequential Organ Failure Assessment (SOFA) [20] score were also measured at the two time points. Also, the need for mechanical ventilation was recorded and the usage of inotropic equivalent was calculated to evaluate the vasopressor dose [21]. Then we measured and recorded patients' outcome including in-hospital mortality and RRT wean-off.

Definitions were made as following: diabetes, previous usage of insulin or oral hypoglycemic agents; hypertension, blood pressure above 140/90 mmHg or usage of anti-hypertension agents; cardiac failure, low cardiac output with a central venous pressure (CVP) above 12 mmHg and an dopamine equivalent above 5 μg/kg/min [21]; chronic kidney disease (CKD), sCr of 1.5 mg/dl or greater documented prior to this admission [22]; sepsis, persisted or progressive signs and symptoms of the systemic inflammatory response syndrome with a documented or presumed persistence of infection [23]; RRT wean-off, cessation from RRT for at least 30 days [15].

The types of major abdominal surgery were further divided into five categories depending on the involvement of abdominal organs: (1) hepatobiliary organ, (2) upper gastrointestinal (GI) tract, (3) lower GI tract, (4) urological organs, and (5) other sites. 'Upper GI tract' was defined as the duodenum and above, while 'lower GI tract' included the area from the jejunum to rectum. If the surgery didn't involve the one of the four major organs (1 to 4), it would be categorized as 'other sites' (5).

The modality of RRT was chosen according to the hemodynamics of the patients. Continuous venovenous hemofiltration was performed, if more than 15 points of inotropic equivalent [15] was required to maintain systemic blood pressure up to 120 mmHg, using high-flux filters (Hemofilter, PAN-10, Asahi Kasei, Japan) and HF 400 (Informed, Geneva, Switzerland). The hemofiltration flow and blood flow blood flow were 35 ml/kg/hour and 200 ml/min, respectively. Replacement fluid was bicarbonate-buffered and was administered predilutionally at a dynamically adjusted rate to achieve the desired fluid therapy goals. Default composition was sodium 142 mEq/l, bicarbonate 33 mEq/l, calcium 2.6 mEq/l, and magnesium 1.4 mEq/l. Intermittent hemodialysis was performed for four hours except for the first and second sessions with a dialysate flow of 500 ml/min and blood flow of 200 ml/min [18], using low-flux polysulfone hemofilters (KF-18C, Kawasumi Laboratories, Shinagawa-ku, Tokyo, Japan). Double lumen catheters were placed as vascular access.

In the ICUs, the indications for RRT initiation were: (1) azotemia (BUN > 80 mg/dL and sCr > 2 mg/dl) with uremic symptoms (encephalopathy, nausea, vomiting, etc); (2) oliguria (urine amount < 200 ml/8 hours) or anuria refractory to diuretics; (3) fluid overload refractory to diuretics use with a CVP level above 12 mmHg or pulmonary edema with a partial pressure of arterial oxygen/fraction of inspired oxygen ratio below 300 mmHg; (4) hyperkalemia (sK^+ ^> 5.5 mmol/L) refractory to medical treatment; and (5) metabolic acidosis (a pH < 7.2 in arterial blood gas) [13,18]. We recorded all the indications of patients upon RRT initiation.

### Covariate

Patients were categorized into two groups (early dialysis (ED) and late dialysis (LD)) according to their RIFLE (Risk, Injury, Failure, Loss, and End stage) classification [24] (Table 1) before RRT initiation. The RIFLE classification was first proposed by the Acute Dialysis Quality Initiative group in an attempt to standardize AKI study, and the scores could be used to predict the mortality after major surgery [25,26]. There were many studies comparing the prognoses among patients in different categories of RIFLE classification, but only a few studies [27,28] compared the outcome among patients who initiated RRT in different categories of RIFLE classification. As in previous studies [27,29,30], we used 'simplified' RIFLE (sRIFLE) classification with only GFR criterion applied for classification because the eight-hourly urine volumes in our database could not match the 6- or 12-hourly urine output criterion in RIFLE classification. Those who initiated RRT when in sRIFLE-R (risk) or sRIFLE-0 [26], which means not yet reaching the sRIFLE-R level were defined as 'ED', while in sRIFLE-I (injury) or sRIFLE-F (failure) were classified as 'LD'. The baseline sCr was the data obtained at hospital discharge from the previous admission in those who had more than one admission [29], or the data estimated using the Modification of Diet in Renal Disease (MDRD) equation [31] in those with only one admission (assuming an average GFR of 75 ml/min/1.73 m^2^). The peak sCr was defined as the highest sCr before RRT initiation in ICUs. The GFR were estimated using the isotope dilution mass spectrometry--traceable four-variable MDRD equation [31].

**Table 1:** RIFLE classification [24] for acute kidney injury

	GFR criteria	Urine output criteria
Risk	Increase plasma creatinine ×1.5 or GFR decrease > 25%	< 0.5 ml/kg/h × 6 h
Injury	Increase plasma creatinine ×2 or GFR decrease > 50%	< 0.5 ml/kg/h × 12 h
Failure	Increase plasma creatinine ×3 or GFR decrease > 75%, or serum creatinine ≥ 4 mg/dL with an acute rise > 0.5 mg/dL	< 0.3 ml/kg/h × 24 h or anuria ×12 h
Loss	Persistent ARF = complete loss of kidney function > 4 wk	
ESRD	End-stage renal disease (> 3 month)	

ARF, acute renal failure; ESRD = end stage renal disease; GFR = glomerular filtration rate; h = hours.

### Outcomes

The endpoint of this study was in-hospital mortality. The survival period was calculated from RRT initiation to mortality (in non-survivors) or hospital discharge (in survivors).

### Statistical analysis

Statistical analyses were performed with the Scientific Package for Social Science for Windows (SPSS, version 13.0, SPSS Inc, Chicago, IL, USA). Continuous data were expressed as mean ± standard deviation unless otherwise specified. Percentage was calculated for categorical variables. Student's *t *test was used to compare the means of continuous data, whereas Chi-squared test or Fisher's exact test was used to analyze categorical proportions. Then we used backward stepwise likelihood ratio model of Cox proportional hazard method to analyze the independent predictors for in-hospital mortality. The independent variables were selected for multivariate analysis if they had a *P *≤ 0.1 on univariate analysis. The basic model-fitting techniques for (1) variable selection, (2) goodness-of-fit assessment, and (3) regression diagnostics (e.g., residual analysis, detection of influential cases, and check for multicollinearity) were used in our regression analyses to ensure the quality of analysis results. Specifically, we used the stepwise variable selection procedure with both significance level for entry and significance level for stay set to 0.15 or larger to select the relevant covariates into the final Cox proportional hazards model. Also, we did an additional analysis adjusting for three clinical relevant variables (namely, sepsis before RRT, mechanical ventilation, and diabetes) regardless of *P *value because they were considered important. Furthermore, we did the analysis comparing sRIFLE categories against each other for the relative risk (RR) for in-hospital mortality. In statistical testing, two-sided *P *value less than 0.05 was considered statistically significant.

Finally, Kaplan-Meier survival curves with log-rank test was drawn to express the differences of patient survival between the two groups (ED versus LD).

## Results

**Figure 1:**
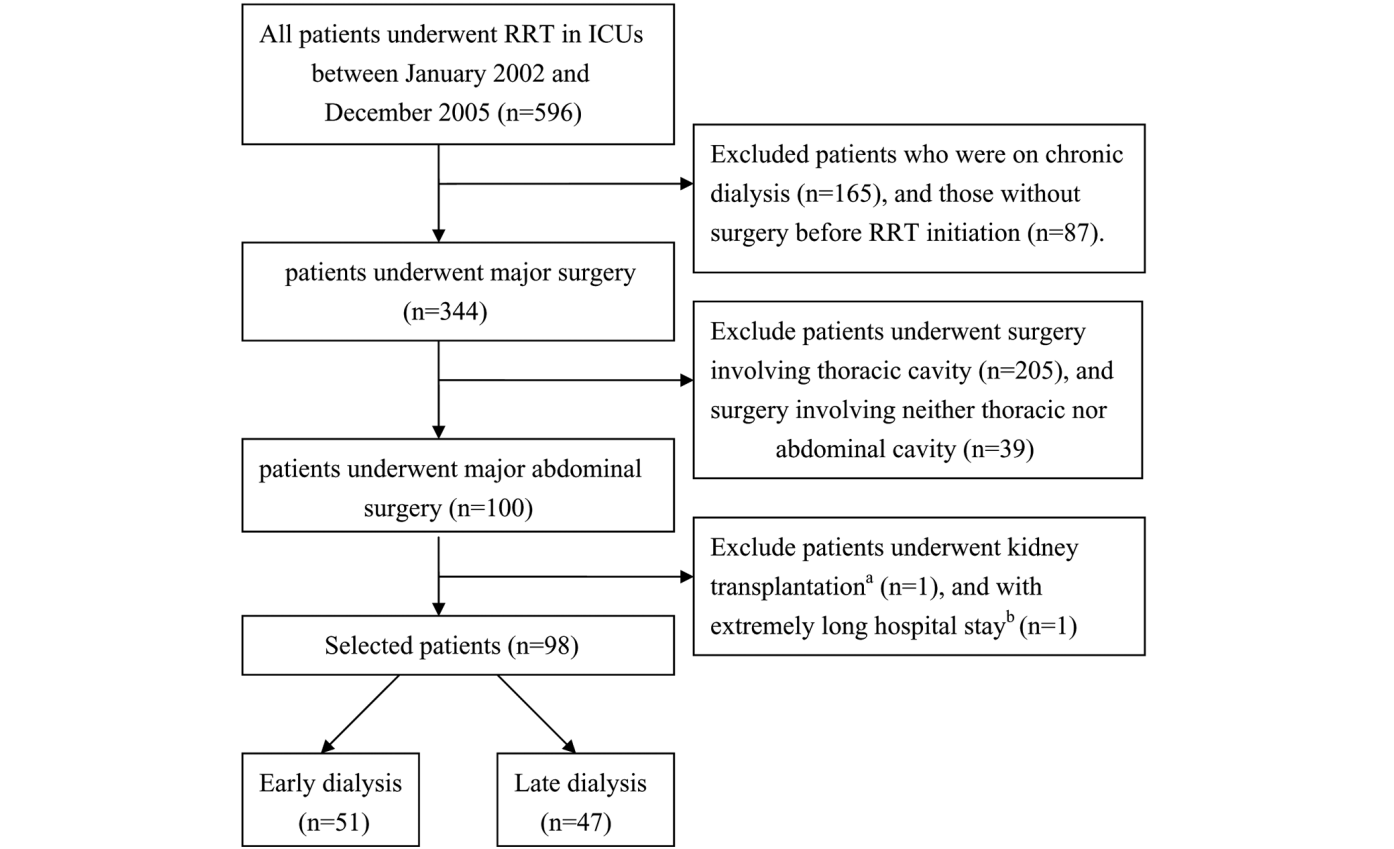
Approach to gathering and selecting patients. ^a^A 44-year-old male received kidney transplantation prior to RRT. ^b^A 85-year-old female whose hospital course is extremely long (727 days from RRT initiation to death, comparing to mean period of 34.3 ± 27.6 days in other 98 patients). ICU = intensive care unit; RRT = renal replacement therapy.

Five hundred and ninety-six patients were screened. Patients on chronic dialysis (n = 165), those without surgery prior to RRT initiation (n = 87), or those whose surgery did not involve abdominal cavities (n = 244) were excluded. A 44-year-old male patient receiving kidney transplantation and an 85-year-old female patient with an extremely long hospital stay period (740 days from ICU admission to death, and 727 days from RRT initiation to death) were also excluded. Figure 1 shows the flowchart of patient gathering and selecting. Finally, a total of 98 patients (41 female, 57 male; mean age 66.4 ± 13.9 years) were selected and followed until 30 June, 2006. Of the 98 patients who underwent acute RRT following major abdominal surgery, most patients (57.1%) underwent elective surgery. Surgery of the hepatobiliary organ was performed in 26 patients (26.5%), upper GI tract in 28 (28.6%), lower GI tract in 29 (29.6%), urological organs in 9 (9.2%), and other sites in 6 (6.1%). The surgery involving the hepatobiliary area included liver transplantation for hepatic failure (n = 14), hepatectomy or lobectomy for hepatoma (n = 4), as well as cholecystectomy or choledocholithotomy owing to CBD stone (n = 3), acute or chronic cholecystitis (n = 4), and gall bladder adenocarcinoma (n = 1). The surgery involving upper GI were gastrotomy, gastrectomy, or simple closure for peptic ulcer bleeding (n = 11), hallow organ perforation (n = 7), and malignancy (n = 4). Also, a Whipple operation for pancreatic cancer (n = 5) and chronic pancreastitis with obstructive jaundice (n = 1) were also categorized as upper GI surgery. The causes of lower GI surgery were colon-rectal malignancy (n = 14), colon perforation (n = 5), exploratory laparotomy for appendicitis and colitis (n = 6), previous operation-related adhesion (n = 2), and ischemic bowel (n = 2). The surgery in urologic organs were nephrectomy, nephroureterectomy, and cystectomy related to malignancy (n = 9). Those included in the 'other sites' category were vein bypass for inferior vena cava occlusion (n = 1), abdominal aortic grafting (n = 1), repair of previous operation wound laceration (n = 1), and exploratory laparotomy for traffic accident (n = 1) and peritonitis (n = 2). The indications for RRT were 42 patients (42.9%) started RRT due to azotemia with uremic symptoms, 40 (40.8%) for oliguria, 10 (10.2%) for fluid overload or pulmonary edema, and 14 (14.3%) for hyperkalemia or acidosis. Because some patients had more than one indication to start RRT, the sum of patient numbers were 106 instead of 98 patients.

Among the 98 patients, 51 patients (52.0%; 22 in sRIFLE-0 and 29 in sRIFLE-R) and 47 patients (48.0%; 27 in sRIFLE-I and 20 in sRIFLE-F) were clarified as ED and LD groups, respectively. Fifty-three patients (54.1%) died during ICU admission (21 (41.2%) in ED group, 32 (68.1%) in LD group), while a total of 57 patients (58.2%) died during their whole hospital course (22 (43.1%) in ED group, 35 (74.5%) in LD group). The LD group has a much lower prevalence of CKD (27.7% versus 54.9%, *P *= 0.008), higher in-hospital mortality rate (74.5% versus 43.1%, *P *= 0.002) and borderline lower RRT wean-off rate (21.3% versus 41.2%, *P *= 0.050) as compared with the ED group. The baseline GFR (60.6 ± 28.5 versus 47.7 ± 27.2, *P *= 0.024) is higher, but baseline sCr (1.3 ± 0.6 versus 2.1 ± 1.7, *P *= 0.003) and pre-RRT GFR (17.5 ± 7.8 versus 32.8 ± 50.3, *P *= 0.036) are lower in the LD group. The differences of other demographic, biochemistry data, severity scores, and usage of diuretics or vasopressors were not statistically significant (Table 2).

**Table 2:** Comparisons of demographic data and clinical parameters between early and late dialysis groups (n = 98)

	Early dialysis (n = 51)	Late dialysis (n = 47)	*P *value
**Demographic data**			
Female	19 (37.3)	22(46.8)	0.414
Diabetes	14 (27.5)	17 (36.2)	0.391
Hypertension	22 (43.1)	20 (42.6)	1.000
Cardiac failure	6 (11.8)	4 (8.5)	0.743
Chronic kidney disease	28 (54.9)	13 (27.7)	0.008
Sepsis before RRT	14 (27.5)	17 (36.2)	0.391
Emergency surgery	23 (45.1)	19 (40.4)	0.686
CVVH	26 (51.0)	31 (66.0)	0.155
Mechanical ventilation	39 (76.5)	40 (85.1)	0.316
Age (years)	65.0 ± 14.8	68.0 ± 13.0	0.284
Old age (> 65 years)	27 (52.9)	30 (63.8)	0.310
Hospital stay (days)	53.7 ± 39.2	54.2 ± 33.6	0.944
Hospital admission to ICU (days)	10.3 ± 15.5	12.6 ± 13.0	0.423
Hospital admission to RRT (days)	17.5 ± 20.3	21.0 ± 19.0	0.388
ICU to RRT (days)	7.3 ± 13.2	8.4 ± 13.6	0.679
RRT to death/discharge (days)	35.5 ± 29.0	33.1 ± 26.2	0.671
Baseline creatinine (mg/dl)	2.1 ± 1.7	1.3 ± 0.6	0.003
Baseline GFR (ml/min/1.73 m^2^)	47.7 ± 27.2	60.6 ± 28.5	0.024
**Operation sites**^a^			0.592
Hepatobiliary system	13 (25.5)	13 (27.7)	
Upper GI	13 (25.5)	15 (31.9)	
Lower GI	15 (29.4)	14 (29.8)	
Urologic system	7 (13.7)	2 (4.3)	
Other sites	3 (5.9)	3 (6.4)	
**Data at ICU admission**			
Diuretics	43 (84.3)	35 (74.5)	0.316
Vasopressors	27 (52.9)	25 (53.2)	1.000
Inotropic equivalent	7.80 ± 12.63	5.96 ± 9.87	0.426
Hematocrit (%)	29.3 ± 7.3	31.5 ± 5.5	0.105
BUN (mg/dl)	50.9 ± 33.0	41.9 ± 27.0	0.143
Creatinine (mg/dl)	2.9 ± 1.9	2.3 ± 1.4	0.137
GFR (ml/min/1.73 m^2^)	39.5 ± 50.0	38.6 ± 26.7	0.917
Albumin (g/dl)	2.8 ± 0.6	2.6 ± 0.7	0.231
Potassium (mEq/l)	4.2 ± 0.7	4.1 ± 0.7	0.552
PaO_2_/FiO_2_	281.1 ± 112.1	273.0 ± 1 20.9	0.732
GCS scores	13.4 ± 3.3	12.6 ± 3.8	0.304
APACHE II scores	18.2 ± 5.4	18.8 ± 6.3	0.620
SOFA scores	8.3 ± 2.7	8.5 ± 3.7	0.767
**Pre-RRT data**			
Hematocrit (%)	28.5 ± 4.8	29.4 ± 5.0	0.374
BUN (mg/dl)	68.8 ± 39.4	81.9 ± 39.3	0.104
Creatinine (mg/dl)	3.3 ± 1.8	3.8 ± 1.3	0.188
GFR (ml/min/1.73 m^2^)	32.8 ± 50.3	17.5 ± 7.8	0.036
Albumin (g/dl)	2.8 ± 0.6	2.8 ± 0.7	0.722
Potassium (mEq/l)	4.2 ± 0.8	4.3 ± 0.7	0.740
PaO2/FiO2	300.3 ± 112.1	280.2 ± 119.5	0.395
GCS scores	12.5 ± 3.9	11.3 ± 4.5	0.160
APACHE II scores	18.2 ± 6.1	20.5 ± 5.8	0.061
SOFA scores	9.4 ± 3.1	10.5 ± 3.8	0.114
**Indications for RRT**			
Azotemia with uremic symptoms^b^	19 (37.3)	23 (48.9)	0.308
Oliguria or anuria^c^	23 (45.1)	17 (36.2)	0.415
Fluid overload or pulmonary edema^d^	4 (7.8)	6 (12.8)	0.513
Hyperkalemia or acidosis^e^	8 (15.7)	6 (12.8)	0.717
**Hospital mortality**	22 (43.1)	35 (74.5)	0.002
**RRT wean-off**	21 (41.2)	10 (21.3)	0.050

Early dialysis group, RIFLE-0 (n = 22) and --R (n = 29); Late dialysis group, RIFLE-I (n = 27) and --F (n = 20).

Values are presented as mean ± standard deviation or number (percentage) unless otherwise stated. ^a^upper GI denotes duodenum and above, lower GI means jejunum and below, other sites denote surgery site besides previous four; ^b ^azotemia was defined as BUN > 80 mg/dL and creatinine > 2 mg/dL; ^c^oliguria was defined as urine output < 200 ml/8 hours refractory to diuretics; ^d^fluid overload means CVP > 12 mmHg, while pulmonary edema denotes PaO_2_/FiO_2 _< 300 mmHg; ^e ^hyperkalemia denotes serum potassium > 5.5 mmol/L, acidosis denotes pH < 7.2 in arterial blood.

APACHE II = Acute Physiology and Chronic Health Evaluation II; BMI = body mass index; BUN = blood urea nitrogen; CVP = central venous pressure; CVVH = continuous venovenous hemofiltration; FiO2 = fraction of inspired oxygen; GCS = Glascow Coma Scale; GFR = glomerular filtration rate; GI = gastrointestinal; ICU = intensive care unit; MAP = mean arterial pressure; PaO2 = partial pressure of arterial oxygen; RRT = renal replacement therapy; SOFA = Sequential Organ Failure Assessment; WBC = white blood cell.

The statistically different demographic data between survivors and non-survivors were age (*P *= 0.014), cardiac failure (*P *= 0.005), sepsis before RRT (*P *= 0.002), length of hospital stay (*P *= 0.025), and the period from ICU and RRT to death or discharge (*P *= 0.005 and < 0.001 respectively). GCS (*P *= 0.040) and APACHE II scores (*P *= 0.010) at ICU admission, and pre-RRT platelet count (*P *= 0.027), BUN (*P *= 0.016), GCS (*P *< 0.001), APACHE II scores (*P *< 0.001), SOFA scores (*P *= 0.005), as well as the percentage of LD (*P *= 0.002) and RRT wean-off rate (*P *< 0.001) were also statistically different. Other comorbid diseases, clinical parameters, and usage of diuretics or vasopressors were not statistically significant as compared between these two groups.

Using the backward stepwise likelihood ratio model of Cox proportional hazard method for in-hospital mortality, LD (hazard ratio (HR) 1.846; 95% confidence interval (CI) 1.071-3.182; *P *= 0.027), old age (older than 65 years) (HR 2.090; 95% CI 1.196-3.654, *P *= 0.010), cardiac failure (HR 4.620; 95% CI 2.216-9.632; *P *< 0.001), and pre-RRT SOFA score (HR 1.152; 95% CI 1.065-1.247; *P *< 0.001) were independent indicators for in-hospital mortality (Table 3). The predictive power for in-hospital mortality of LD (HR 1.756; 95% CI, 1.003-3.074; *P *= 0.049) persisted in the additional Cox regression analysis in which the three variables (sepsis before RRT, mechanical ventilation, and diabetes) was forced into the analysis regardless of *P *value. From the analysis comparing 'sRIFLE' categories against each other, we found a significant RR of 'sRIFLE-F' (RR 3.194, *P *= 0.014), and a trend of increased risk of 'sRIFLE-I' (RR 2.121, *P *= 0.080) as comparing with 'sRIFLE-R' (Table 4).

**Table 3:** Independent predictors for in-hospital mortality using Cox proportional hazards model

Variables	Univariate			Multivariate (Backward stepwise likelihood ratio)		
		
	HR	95% CI	*P*	HR	95% CI	*P*
Old age (> 65 years)^a^	1.960	1.127--3.408	0.017	2.090	1.196--3.654	0.010
Cardiac failure^b^	4.084	2.003--8.328	< 0.001	4.620	2.216--9.632	< 0.001
Pre-RRT SOFA score^c^	1.138	1.054--1.228	0.001	1.152	1.065--1.247	< 0.001
CVVH^d^	1.940	1.123--3.352	0.018	----	-----	-----
Late dialysis^e^	1.852	1.081--3.170	0.025	1.846	1.071--3.182	0.027

The independent variables were selected for multivariate analysis if they had a *P *≤ 0.1 on univariate analysis.

Data were gathered before RRT initiation. Duration in analysis is calculated from RRT initiation to end point (mortality or discharge).

^a ^hazard for patients > 65 years = 1.0; ^b ^hazard for patients without cardiac failure = 1.0; ^c ^every increment of 1 point; ^d ^hazard for patients underwent intermittent hemodialysis = 1.0; ^e ^Late dialysis denotes initiation RRT in RIFLE-R and -F, hazard for patients in early dialysis group (start RRT in RIFLE-0 and --I) = 1.0.

APACHE II = Acute Physiology and Chronic Health Evaluation II; CVVH = continuous venovenous hemofiltration; HR = hazard ratio; 95% CI = 95% confidence interval; RRT = renal replacement therapy; SOFA = Sequential Organ Failure Assessment.

**Table 4:** Relative risk (RR) for in-hospital mortality using Cox proportional hazards model

RIFLE categories	Patient number (%)	RR*	95% CI	*P*
RIFLE - R	29 (29.6)	1.000	Reference	
RIFLE - I	27 (27.6)	2.121	0.913-4.927	0.080
RIFLE - F	20 (20.4)	3.194	1.262-8.085	0.014

* Adjusted for age (≧ 65 years vs < 65 years), cardiac failure (with vs without), pre-RRT SOFA scores, and RRT modality (CVVH vs hemodialysis); CVVH = continuous venovenous hemofiltration; 95% CI = 95% confidence interval; RR = relative risk; RRT = renal replacement therapy; SOFA = Sequential Organ Failure Assessment.

**Figure 2:**
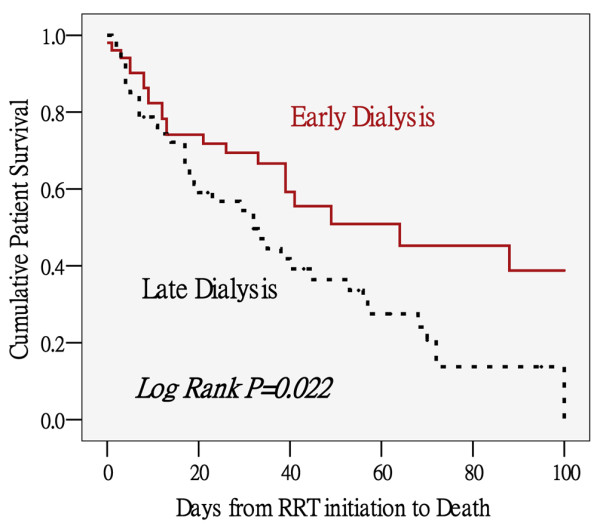
Cumulative patient survival between early and late dialysis groups defined by RIFLE classification. By Kaplan-Meier method. Brown solid line = early dialysis group (RIFLE-0 and -I, n = 51); black dashed line = late dialysis group (RIFLE-R and -F, n = 47). RRT = renal replacement therapy.

By Kaplan-Meier curves, we demonstrated that the survival proportion was much lower in LD group as compared with ED group (*P *= 0.022; Figure 2).

## Discussion

### RIFLE classification and RRT initiation

The RIFLE classification [24] was proposed to standardize the severity of AKI, and it's predictive value for patient outcome was supported by many studies [25,26,32]. The stratification about the timing of RRT initiation by RIFLE classification has been recommended by the Acute Kidney Injury Network [33]. Our work is among the first few studies examining the relation between prognosis and timing of RRT initiation. We found that late initiation of RRT as defined by 'sRIFLE-I' and 'sRIFLE-F' is an independent predictor for in-hospital mortality in a relative homogenous group of patients with AKI after major abdominal surgery.

### Early versus late initiation of RRT

Current practice suggests that RRT is indicated for a patient with an abruptly decreased renal function along with clinically significant solute imbalance or volume overload, yet there is no consensus on the definite indication for RRT in terms of any single metabolic or clinical parameters or RIFLE staging [33]. Although the benefit of early RRT initiation on survival outcome was revealed by a recent systemic review and meta-analysis [9], the question of 'how early is early enough?' is still unanswered because the early versus late RRT were defined by variable cutoff values of various metabolic parameters such as nitrogenous waste products, sCr, sK^+ ^[34], urine amount, or even clinical judgment alone [9,35]. The present study defines the timing of RRT initiation by using RIFLE classification because this has been extensively validated to standardize the severity of AKI [33].

As it is reasonable that the patient survival is artificially extended if it is measured at an earlier time point with better residual renal function and less severity scores, and the so-called survival benefit from early RRT could be accounted for by lead-time bias [34]. However, the period from hospital admission to RRT initiation, as well as the severity scores including APACHE II score and SOFA score and almost all clinical parameters upon RRT initiation, which was taken as a starting point to calculate survival period, were of no statistical differences between ED and LD groups (Table 2). Therefore, the argument of lead-time bias would be minimized in the current study.

Two recent published studies [27,28] have evaluated the association between the timing of RRT initiation by the RIFLE classification and outcome. Neither of them propose clearly defined indications for RRT. Only 33% patients in one study [28] and none in the other [27] were categorized using both GFR and urine output criteria. The retrospective observational study by Li and colleagues [27] enrolled 106 critical AKI patients treated with continuous RRT. It found that the RIFLE classification may be used to predict 90-day survival after RRT initiation, and further analysis revealed that patient in RIFLE-F had a RR of 1.96 (95% CI: 1.06-3.62) comparing with those in RIFLE-R. The predictive effect was also seen in our work in which the RR of sRIFLE-F to sRIFLE-R was 3.194 (*P *= 0.014). As to the study of Maccariello and colleagues [28], a prospective cohort study including 214 AKI patients who underwent RRT, the RIFLE classification didn't show discrimination of prognosis in all patient populations. However, the association between RIFLE-F and increased in-hospital mortality was found while conducting a separate analysis study using only patients who underwent ventilation and vasopressors.

### Indications of RRT initiation

In our study, RRT was provided to the patients according to the five criteria, namely, (1) azotemia with uremic symptoms, (2) oliguria or anuria, (3) fluid overload, (4) hyperkalemia, and (5) metabolic acidosis. Although the criteria for RRT were not too loose compared with those in other studies [9,33], about half of the patients who underwent RRT (n = 51, 52.0%) were categorized into the ED group. This result was not surprising when compared with the largest study on the epidemiology of AKI during the entire ICU stay by Ostermann and Chang [30]. Among the total 1847 patients who underwent RRT in that study, only 573 (31.0%) fulfilled the sCr criterion and 691 (37.4%) would probably fulfill the urine criterion for AKI stage III, and the remaining 583 (31.6%) would be classified into earlier stage [36]. Actually, RIFLE classification and our own criteria for RRT are different scoring systems. The numbers of our indications for RRT are more than the parameters used in the RIFLE classification (only sCr level, GFR, and urine amount). Although the parameters in the RIFLE classification seems similar to the former two of our five RRT indications, the percentage change in sCr or GFR in RIFLE classification was different from the absolute BUN or sCr level in ours. Furthermore, 'oliguria or anuria' played a significant role as an indication for RRT in our study (45.1% and 36.2% in ED and LD, respectively) (Table 2), but the urine criterion of RIFLE classification was not used in categorizing patients. It means that those who met our study indications and received RRT accordingly may not be considered serious by RIFLE classification.

In critically ill patients, AKI is usually associated with multiple-organ failure. Preventing further renal damage and recovering renal function are largely dependent on recovery of other organ function. Thus, the concept has changed from 'renal replacement' to 'renal support' in ICU patients [37-39]. However, RRT has often been applied too late [40], leading to prolonged and poorly controlled uremia, restricted nutrition, acidosis, and volume overload [41]. In this study, the indications for RRT were not statistically different between ED and LD groups (Table 2), and survivors and non-survivors (detail not shown in the text). Thus, the survival benefit could not be simply explained by the causes of RRT initiation (such as fluid management or toxin removal), and the importance of early initiation of RRT clearly speaks for itself in this study [9].

### Predictors for in-hospital mortality

More than half of patients who underwent RRT following major abdominal surgery died during hospital admission, which is comparable with previous studies [29,42,43]. Our study found that LD defined by sRIFLE classification, along with old age, cardiac failure, and pre-RRT SOFA scores, are strong predictors for in-hospital mortality. Old age has been a well-recognized predictor for mortality in critically ill surgical patients in many studies [28,43,44]. Cardiac failure is not only characterized by a high rate of hospital readmission and mortality in the general population [45], but is also considered an independent predictors for mortality in critically ill surgical patient with AKI [43]. Besides, SOFA score was chosen as a representation of severity score for Cox analysis in our study. The predictive value for poor prognosis in AKI of SOFA score has been reported in other studies [1,30] as well.

Similar to the report of a systemic review and meta-analysis summarizing all studies published before 2008 [9], our data supported the survival benefit in earlier initiation of RRT. However, discordant results existed. Bagshaw and colleagues [46] designed a prospective multicenter observational study enrolling 1238 patients to evaluate the relation between timing of RRT initiation in severe AKI and prognoses. Timing of RRT was assessed by several approaches such as median value and median change of BUN and sCr, and the period from ICU admission to start of RRT. Contrary to our findings, they found late RRT stratified by median sCr was associated with lower mortality. Previous studies [47] using sCr criterion to define early RRT also failed to show survival benefit. The main plausible explanation is that low sCr levels might not necessarily represent a better residual renal function. In contrast, the low sCr could be a marker of reduced muscle mass and malnutrition, and it may be a surrogate marker of volume overload, which in turn might contribute to poor survival [33,46].

However, this bias did not exist in our study because the sCr and albumin level were not statistically different between ED and LD groups upon ICU admission and before RRT initiation (Table 2). In fact, the relation between sCr and mortality was ever documented to be paradoxical in dialysis patients, which is called 'reverse epidemiology'. It refers to paradoxical and counter-intuitive epidemiologic associations between survival outcomes and traditional risk factors such as creatinine [48].

It is worthy of mention that the LD group in our study has better baseline renal function (less CKD proportion, lower baseline sCr, higher baseline GFR) but worse pre-RRT renal function. There is no doubt that a larger sCr increase or GFR decrease categorized patients into LD group, but it also gave a hint that those with more sever renal function deterioration have poorer outcome. Actually, both the proportional change of sCr or GFR in RIFLE classification, and the absolute sCr level in the SOFA scores could predict prognoses in our patients. This finding was supported by Coca and colleagues [49] who had disclosed the prognostic importance of a small acute change in sCr in absolute level as well as percentage changes.

### Limitations and summary

Several limitations for this study should be recognized. First, the limited patient number may not be large enough to determine other risk factors for in-hospital mortality. Second, only GFR criterion of RIFLE classification was used in the current study. Although several studies [29,30] did the same, it is a shortcoming to lack urine output when applying the RIFLE category. Thus we used the term 'sRIFLE' in our manuscript to distinguish from the original RIFLE. Therefore, observations accrued here might not be extrapolated to patients with AKI elsewhere. Further multicenter randomized clinical trials are warranted to confirm our findings.

## Conclusions

LD defined by RIFLE-I or RIFLE-F of 'simplified' RIFLE classification is an independent predictor for in-hospital mortality in the current study. Our findings support earlier initiation of RRT, and also underscore the importance of predicting prognoses of patients with AKI by using RIFLE classification.

## Key messages

• AKI is a common problem in critically ill patients, and postoperative AKI is one of the most serious complications in surgical patients.

• The RIFLE classification was proposed to standardize AKI study, and it's predictive value for patient outcome was supported by many studies.

• Late initiation of RRT defined by RIFLE-I or RIFLE-F is an independent predictor for in-hospital mortality in the current study. Our findings support early initiation of RRT, and also underscore the importance of predicting prognoses of patients with AKI by using RIFLE classification.

## Abbreviations

AKI: acute kidney injury; APACHE II: Acute Physiology and Chronic Health Evaluation II; BUN: blood urea nitrogen; CI: confidence interval; CKD: chronic kidney disease; CVP: central venous pressure; ED: early dialysis; GCS: Glascow Coma Scale; GFR: glomerular filtration rate; GI: gastrointestinal; HR: hazard ratio; ICU: intensive care unit; LD: late dialysis; MDRD: Modification of Diet in Renal Disease; RR: relative risk; RRT: renal replacement therapy; sCr: serum creatinine; sK^+:^serum K; SOFA: Sequential Organ Failure Assessment.

## Competing interests

The authors declare that they have no competing interests.

## Authors' contributions

CCS, VCW, and CCK have made substantial contributions to conception and design, and drafted the manuscript. WYL, DMH, SLL and PRT were involved in acquisition and interpretation of data. YFL, GHY, and CHW participated in the sequence alignment and drafted the manuscript. FCH, NKC, and THL participated in the design of the study and performed the statistical analysis. TWK, YCY, and YMC participated in its design and coordination and helped to draft the manuscript. MTL, AC, WJK, and KDW revised the manuscript critically for important intellectual content, and have given final approval of the version to be published. All authors read and approved the final manuscript
